# Development of the digital retrieval system integrating intelligent information and improved genetic algorithm: A study based on art museums

**DOI:** 10.1371/journal.pone.0305690

**Published:** 2024-06-25

**Authors:** Cun Lin, XiaoChen Hu, TianYi Cheng, Rao Yin

**Affiliations:** School of Design and Innovation, China Academy of Art, Hangzhou, Zhejiang, China; Ariel University, ISRAEL

## Abstract

This study aims to develop a digital retrieval system for art museums to solve the problems of inaccurate information and low retrieval efficiency in the digital management of cultural heritage. By introducing an improved Genetic Algorithm (GA), digital management and access efficiency are enhanced, to bring substantial optimization and innovation to the digital management of cultural heritage. Based on the collection of art museums, this study first integrates the collection’s images, texts, and metadata with multi-source intelligent information to achieve a more accurate and comprehensive description of digital content. Second, a GA is introduced, and a GA 2 Convolutional Neural Network (GA2CNN) optimization model combining domain knowledge is proposed. Moreover, the convergence speed of traditional GA is improved to adapt to the characteristics of cultural heritage data. Lastly, the Convolutional Neural Network (CNN), GA, and GA2CNN are compared to verify the proposed system’s superiority. The results show that in all models, the sample output results’ actual value is 2.62, which represents the real data observation results. For sample number 5, compared with the actual value of 2.62, the predicted values of the GA2CNN and GA models are 2.6177 and 2.6313, and their errors are 0.0023 and 0.0113. The CNN model’s predicted value is 2.6237, with an error of 0.0037. It can be found that the network fitting accuracy after optimization of the GA2CNN model is high, and the predicted value is very close to the actual value. The digital retrieval system integrated with the GA2CNN model has a good performance in enhancing retrieval efficiency and accuracy. This study provides technical support for the digital organization and display of cultural heritage and offers valuable references for innovative exploration of museum information management in the digital era.

## Introduction

With the rapid development of information technology, the digital age has greatly changed how people access and share information. In this digital wave, the digital retrieval system, a vital way to obtain information, has become an indispensable part of people’s daily lives [1]. As a treasure house of cultural heritage, art museums have a wide variety of artworks and actively participate in the wave of digitalization. However, due to the complexity and diversity of artwork information, traditional keyword retrieval methods have been unable to meet users’ demands for accurate, efficient, and personalized information retrieval [2]. Intelligent information processing technologies, such as image recognition, natural language processing, and user interest modeling, have shown powerful capabilities in many fields, bringing new possibilities for information retrieval [3]. Nevertheless, how to apply these techniques effectively in the retrieval of artistic works is still a problem worth exploring [4].

With the continuous development of digital technology, the demand and importance of art museum digitalization are becoming increasingly prominent. However, the traditional digital retrieval system often faces some challenges in the face of a large amount of information about artworks, such as large and messy information, semantic understanding difficulty, and performance and efficiency challenges. In this context, this study explores how to integrate intelligent information processing technology and improved GA into the development of an art museum digital retrieval system, to improve users’ retrieval experience and efficiency. By introducing the method of integrated intelligent information processing technology and improved GA, and elaborating the design and implementation process of the system, this study provides a new idea and method for applying the digital retrieval system in the art field. By building a system prototype that integrates a variety of intelligent technologies and optimization algorithms, and verifying the system’s performance and effect through experimental results and analysis, this study contributes to the development of the digital retrieval system field. Therefore, the problem of this study is how to apply intelligent information processing technology and improved GA to an art museum digital retrieval system. Its goal is to improve the user’s search experience and efficiency, which has important theoretical and practical significance.

## Literature review

Genetic Algorithm (GA), as an optimization algorithm, has exhibited excellent performance in the process of solving complex problems. Optimizing the search ranking algorithm in the digital retrieval system can remarkably affect the user’s retrieval experience. Therefore, this study introduces an improved GA, which aims to better satisfy users’ search intention by optimizing search ranking. Many researchers have studied and discussed this issue. Hafsa et al. (2022) proposed a GA for image reconstruction using a non-blind search method that considered prior knowledge about possible conductivity distributions in the initial search space. The algorithm’s performance was evaluated regarding image quality and processing time and minimized the corresponding quality function to 0.0505 in 100 generations using non-blind search and uniform crossover/random variation. Compared with the traditional method, GA achieved significantly better image quality. It had been implemented as an image reconstruction algorithm for gesture recognition [5].

Cao (2021) constructed a three-dimensional (3D) art design system based on dynamic image detection and GA. The system simulated the actual defog method and proposed to use of bilateral filtering instead of median filtering. Because bilateral filtering has good edge retention, it can eliminate the block caused by median filtering. In addition, the research also used the Fast Marching Method (FMM) algorithm to repair the image. To verify the model’s performance, quantitative evaluation was conducted using system simulation and a user satisfaction survey. The research results denoted that the proposed method had certain effects and could be applied to the actual situation [6]. Acharya & Kumar (2021) introduced a new adaptive image enhancement technology: Genetic Algorithm-based Adaptive Histogram Equalization (GAAHE). The framework included GA, histogram segmentation, and an improved probability density function. A new subdivision method was applied to histograms, employing exposure thresholds and optimal thresholds to maintain brightness and reduce information loss. To make the introduced technology more adaptive, the threshold parameters were optimized using the GA concept under the guidance of the proposed multi-objective fitness function. Then, each sub-histogram’s Portable Document Format (PDF) was modified to improve the image quality. Experimental results indicated that the proposed GAAHE was superior to existing enhancement technologies [7].

Some researchers discussed the digital retrieval system. Wu et al. (2022) extended the Technology Acceptance Model (TAM), took information quality (IQ) and information richness (IR) as system characteristics, and implemented a research model. In addition, they proposed 11 assumptions about users’ behavioral intentions toward digital clothing museums. Data analysis of 265 apparel-related respondents showed that IQ positively influenced Perceived Convenience (PC) and Perceived Ease of Use (PEOU). IR had a positive impact on Perceived Usefulness (PU) and Perceived Playfulness (PP). The results also suggested that PU and PP were important predictors of user behavior. The research conclusions enriched academic theories and inspired managers, curators, and practitioners to build and innovate digital costume museums [8]. Chen et al. (2020) introduced a personalized query suggestion diversification model, in which a user’s long-term search behavior was injected into a basic greedy query suggestion diversification model, considering the user’s search context in the current session. The query aspect was identified by click-through documents based on open directory projects that adopted the underlying Dirichlet distribution topic model, using the most recent public America Online query log baseline. The findings demonstrated that the model outperformed the baseline regarding ranking and diversity of query recommendations. Experimental results also illustrated that utilizing queries with only click-through documents as search context could achieve the best performance, especially in lists with more query suggestions [9].

In their latest research, Ross et al. (2024) investigated the surface mechanisms and tool wear indicators after processing additive manufactured 316 L stainless steel under different cooling conditions. The study findings suggested that the use of hybrid cooling techniques could significantly reduce lateral tool wear, providing a promising solution for machining additive-manufactured steel components for aerospace applications [10]. Chauhan et al. (2024) proposed an intelligent technology-based approach, utilizing artificial intelligence algorithms to predict the frictional force of Ti-6Al-4V alloy under various lubrication conditions to assess its friction behavior. The research results indicated that this approach could effectively predict wear and provide strong support for material selection in complex mechanical applications [11]. Vashishtha and Kumar (2022) explored the integration of the Evolutionary Algorithm (EA) and Slime Mould Algorithm (SMA) to enhance the efficiency of global optimization and traditional design problems. By blending these two algorithms in parallel and serial ways, a novel method was created. In the parallel structure, EA and SMA were executed simultaneously, and their solutions were combined to obtain the optimal solution, which was then updated to the global optimal solution. In the serial structure, EA first obtained the optimal solution, which was then passed on to SMA to acquire the global optimal solution. This hybrid approach improved the global search capability and search efficiency of EA and SMA. The study also validated this method’s effectiveness by testing the Parallel Series Evolutionary Algorithm Slime Mould Algorithm (PSEASMA) and Serial Series Evolutionary Algorithm Slime Mould Algorithm (SSEASMA) in the classical benchmark function and Competition on Evolutionary Computation (CEC) 2019 function, using the Wilcoxon rank-sum test. The research results demonstrated that this method outperformed other well-known metaheuristic algorithms in terms of both mean and standard deviation, showing significantly superior performance [12]. Chauhan et al. (2024) described and diagnosed the health condition of Pelton turbine blades, proposing a method based on improved Shannon entropy and Expectation Maximization Principal Component Analysis. The research results denoted that this method had notable advantages regarding diagnostic accuracy and computational efficiency, affording a new technical approach to turbine fault detection [13]. Chauhan and Vashishtha (2023) proposed a novel bearing fault diagnosis method using Singular Value Neutron Cross Entropy to detect bearing defects. By combining artificial intelligence algorithms and feature pattern decomposition techniques, this method achieved accurate diagnosis of bearing defects, offering an effective solution for the industrial mechanical system’s health monitoring [14].

In summary, significant progress has been made in the application of advanced manufacturing technology and intelligent techniques in mechanical engineering, covering various aspects such as material processing and mechanical system fault diagnosis. These research findings provide important references and technical support for improving the quality and performance of mechanical engineering products, reducing failure rates, and maintenance costs. However, there are still some challenges and unresolved issues. For instance, how to further improve prediction accuracy and diagnostic precision, and how to reduce the cost and complexity of technology applications. Therefore, future research needs to further explore and innovate to meet the growing demands and challenges in the mechanical engineering field. The relevance between the aforementioned studies and this study primarily lies in the application of intelligent technology. This study aims to fill the gap mainly in the digital retrieval system’s development and application. Although digital technology has been widely used in art museums, there are still challenges in the design and optimization of retrieval systems. Traditional retrieval systems may suffer from inefficiency and low accuracy. However, leveraging intelligent information technology and improved GA to optimize retrieval systems can enhance the quality and response speed of search results, better meeting user needs. Hence, the main objective of this study is to develop an efficient and accurate digital retrieval system in the field of art museums, addressing the deficiencies in existing systems in terms of intelligence, to enhance user experience and information retrieval efficiency.

## Digital retrieval system integrating improved GA and intelligent information

### Genetic Algorithm 2 Convolutional Neural Network (GA2CNN) optimization model

**Fig 1:**
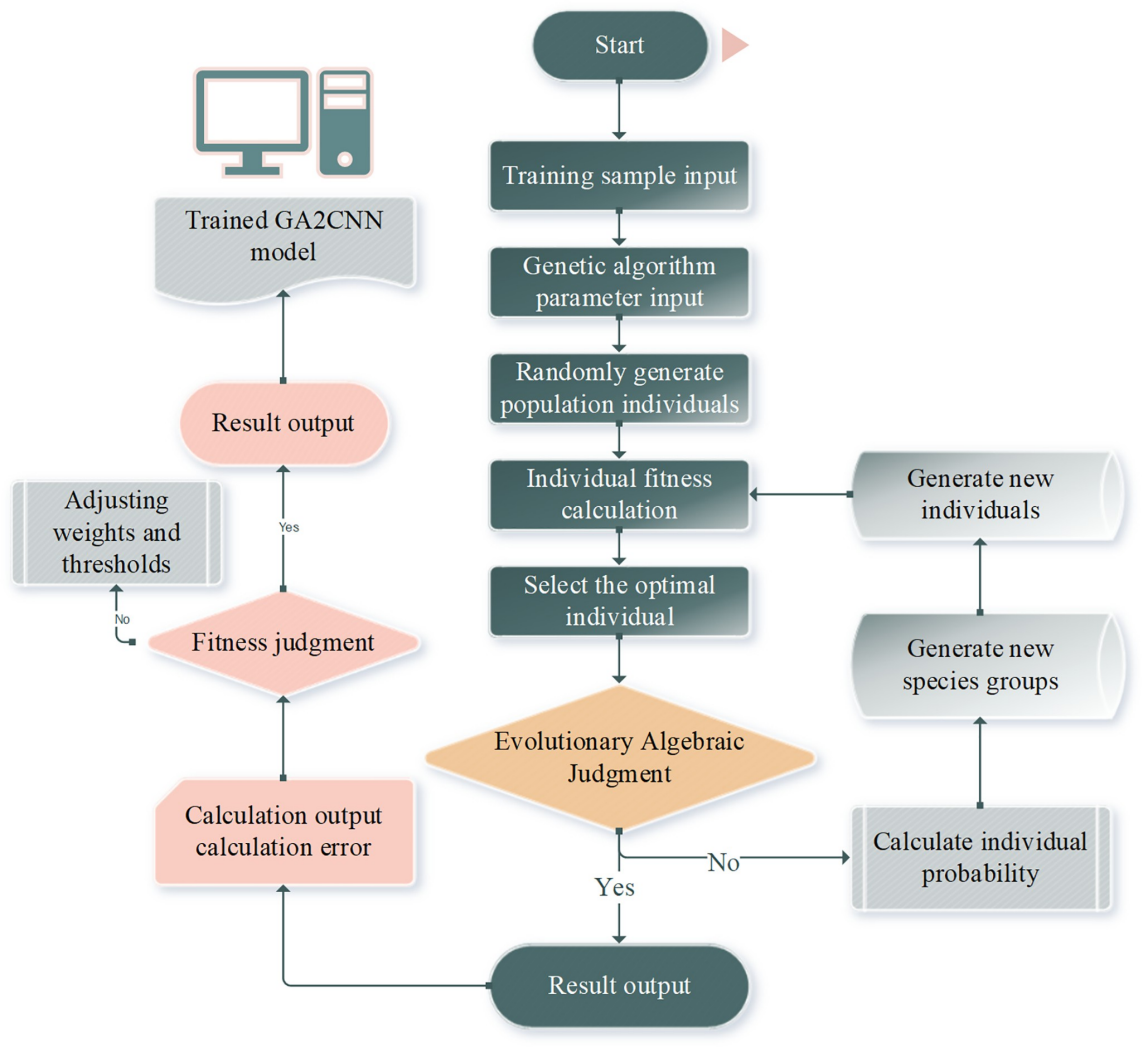
GA2CNN optimization model.

Optimizing CNN based on GA can greatly improve the accuracy and efficiency of evaluation or prediction models. Therefore, the prediction model constructed in this section is based on CNN and GA and adopts the modeling idea of combining GA and CNN. GA consists of 7 steps: (1) Coding. It is mainly to determine the code system and form the genetic code chain. (2) Initialization. The initial population is randomly selected, and the population size is generally 30–160. (3) Fitness estimation. Everyone’s fitness is calculated, individuals with higher fitness representing higher quality solutions. It is also called regeneration, that is, selecting individuals who are better adapted to the environment to reproduce the next generation, and the higher the fitness, the more the number of next-generation individuals. (5) Crossover. The next generation of selected individuals randomly exchanges corresponding genes with a certain probability of producing new individuals. (6) Variation. An individual is randomly selected to perform the mutation with a certain probability. Thus, the homogeneous population can also produce new individuals. (7) Optimal convergence. If the fitness of the optimal individual is lower than the expected threshold or the fitness continues to rise. In other words, if the optimal solution is not obtained, then return to step (3) and perform the cycle until the optimal solution is generated and the algorithm ends. To fully leverage the GA and CNN algorithms’ advantages and improve the prediction accuracy of digital library resource aggregation quality, this study constructs a GA2CNN prediction model, as displayed in Fig 1.

**Fig 2:**
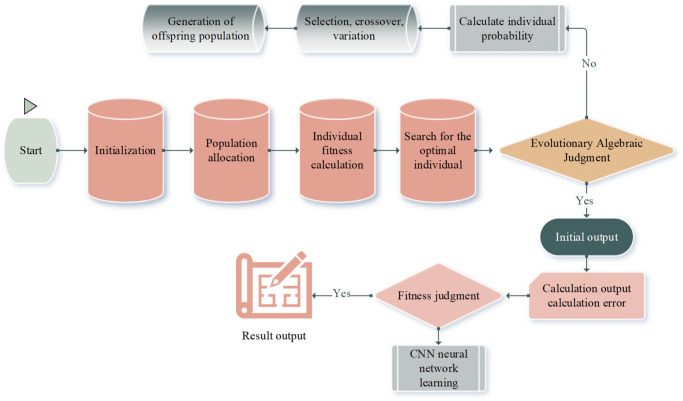
The framework of the GA2CNN model algorithm.

The basic idea of the model is as follows. Firstly, in light of the training sample data, the GA is utilized to search the edge of the global best. Secondly, the CNN algorithm is used for local search to solve the global best. Finally, the model is tested by inputting test data based on the trained model. The framework diagram of the model algorithm is shown in Fig 2.

First, according to the training sample data, GA is employed to find the edge of the global optimal advantage. Then, the CNN algorithm is used for local search to solve the global best of the whole. Lastly, input validation data is used to verify the model according to the trained model. The key execution process and algorithm of the model are described as follows:

In the beginning, the main task is to design the code, build the individual structure, and determine the number of the input, hidden, and output layers. Meanwhile, it is necessary to confirm the number of nodes in each layer [15]. The coding method usually adopts binary coding, the number of input and output layer nodes ***m*** and ***n*** can be determined by the number of units in the problem to be solved, while the number of hidden layer nodes ***I*** adopts trial and error. The determination of the initial value is represented in Eq (1):

$$I=\sqrt{m+n}+\alpha$$ (1)

The initial values in Eq (1) are provided to establish a starting point, making the trial-and-error process more effective. While the number of hidden layer nodes can be determined through trial and error, the choice of initial values is crucial for initiating the trial. The initial values are determined using the number of input layer nodes, ***α***, and the number of output layer nodes. ***α*** can be considered as an adjustment parameter that can influence the speed and direction of the trial-and-error process. The purpose of selecting initial values is to make this process more efficient, thereby finding the appropriate number of hidden layer nodes in as few attempts as possible. Once the initial values are determined, the trial-and-error process can be adjusted based on actual conditions, trying different numbers of hidden layer nodes and evaluating their performance in terms of network performance. Therefore, although the final determination of the number of hidden layer nodes is achieved through trial and error, choosing a suitable initial value can offer a reasonable starting point in this process and help find the optimal number of hidden layer nodes more quickly in a limited number of attempts.

Normalization of training sample data is performed to eliminate the influence of different dimensions, making data processing more robust and effective. The equation for normalizing sample data is as follows:

$$x_{i}=\frac{x_{i}-x_{min}}{x_{max}-x_{min}}$$ (2)

***x***_***i***_ stands for input and output data; ***x***_***max***_ and ***x***_***min***_ represent the maximum and minimum values in the data group. Input parameters for the GA include population size, evolution generations, crossover mutation probability, etc. To improve algorithm accuracy and ensure population diversity, population individuals are represented by the following equation:

$$H_{m}\left(p_{i},p_{i}\right)=\frac{1}{2n+1}\sum_{p=1}^{2n+1}\;H_{g}(g_{i}^{p},g_{j}^{p})$$ (3)

***p***_***i***_ represents the ***i***-th individual of the population, and $H_{g}(g_{i}^{p},g_{j}^{p})$ means the coding position of individuals ***i*** and ***j***. In GA, the term "encoding position" refers to the position of an individual on the chromosome. A chromosome is a structure composed of genes, with each gene corresponding to a feature or attribute of an individual. In GA, these genes are generally represented using binary encoding. The encoding position refers to the index position of genes on the chromosome, such as the first gene, the second gene, and so on. In a population, each individual has its chromosome, and the encoding position is used to indicate the specific position of genes on the chromosome. The hidden and output layers’ output ***s***_***j***_ and ***L***_***t***_ are expressed in Eq (4):

$$s_{j}=f\left(\sum_{j=1}^{m}\;\omega_{ij}x_{i}-\theta_{j}\right)$$ (4)

$$L_{t}=f\left(\sum_{j=1}^{n}\;\nu_{jt}s_{j}-\gamma_{t}\right)$$ (5)

***ω***_***ij***_ and ***ν***_***jt***_ indicate the connection weight of the input-hidden layer and the hidden-output layer; ***θ***_***j***_ and ***γ***_***t***_ refer to the output threshold of the hidden and output layers [16]. Based on the principle of root mean square error minimum and GA requirements, the error ***D***_***t***_ is converted into a maximum fitness function ***F***_***t***_, and the fitness and error calculation read:

$$D_{t}=\frac{1}{\varphi }\sum_{t=1}^{\varphi }\sqrt{{\left(L_{t}-S_{t}\right)}^{2}}$$ (6)

$$F_{t}=\frac{1}{D_{t}}=\frac{\varphi }{\sum_{t=1}^{\varphi }\sqrt{{\left(L_{t}-S_{t}\right)}^{2}}}$$ (7)

***φ*** means the number of samples; ***S***_***t***_ represents the expected output of ***t***-samples. Calculating individual probabilities and generating new individuals is an essential step [17]. At this stage, the probability of each individual is first calculated, and then a new individual is generated. Each individual’s adaptation probability of chromosomes and the cumulative probability are calculated. These probabilities are determined based on their performance in the fitness function and are intended to quantify how good or bad an individual is. The probability of adaptation refers to the probability of an individual’s chromosomes behaving under the fitness function, while the cumulative probability considers the accumulation of the probability of adaptation in all individuals.

These probability values provide the basis for selecting individuals in subsequent steps. In accordance with these probability values, individuals are selected using random selection. Then, chromosomal crossing and mutation operations are performed to form a new population. This probability-based operation can preserve the characteristics of excellent individuals and introduce new variations to increase the diversity of the population, thereby promoting the evolution of the population. During the algorithm execution, the resulting new population is fed back to Eq (3), repeating the entire process. This iterative process continues until a predetermined evolutionary algebra is reached. Predetermined evolutionary algebra refers to a predetermined number of algebras or iterations of evolution in GA. During the execution of GA, the population is evolved according to crossover, mutation, and other operations, and each evolutionary operation is called a generation. The proposed algebra is set before the GA is run, specifying how many generations the algorithm is evolved. When a predetermined evolutionary algebra is reached, the GA stops running and outputs the final result. The setting of this algebra is adjusted based on the problem complexity, the algorithm performance, and other factors. Ultimately, the algorithm outputs initial value parameters such as ***ω***_***ij***_, ***ν***_***jt***_, ***θ***_***j***_, and ***γ***_***t***_. In general, through this process of GA, it is possible to gradually optimize the population with each generation to obtain a set of individual parameters that perform better under the fitness function.

Then, the error and fitness are calculated again. If the accuracy requirements are not met, the weights and thresholds are adjusted according to the following equations:

$$v_{jt}\left(n+1\right)=v_{jt}\left(n\right)+\alpha D_{t}s_{j}$$ (8)

$$\gamma_{t}\left(n+1\right)=\gamma_{t}\left(n\right)+\alpha D_{t}$$ (9)

$$\omega_{tj}\left(n+1\right)=\omega_{tj}\left(n\right)+\alpha D_{j}$$ (10)

$$\theta_{j}\left(n+1\right)=\theta_{j}\left(n\right)+\alpha D_{t}$$ (11)

The purpose of updating the weights multiplied by ***D***_***t***_***s***_***j***_ is to adjust the weights based on the product of the error and the output of the hidden layer, which is called gradient descent. Until the sample training is completed, error ***D*** meets the accuracy requirements, and the learning is over. That is, the network converges and outputs the optimal value.

### Design of the digital retrieval system of the art museum

**Fig 3:**
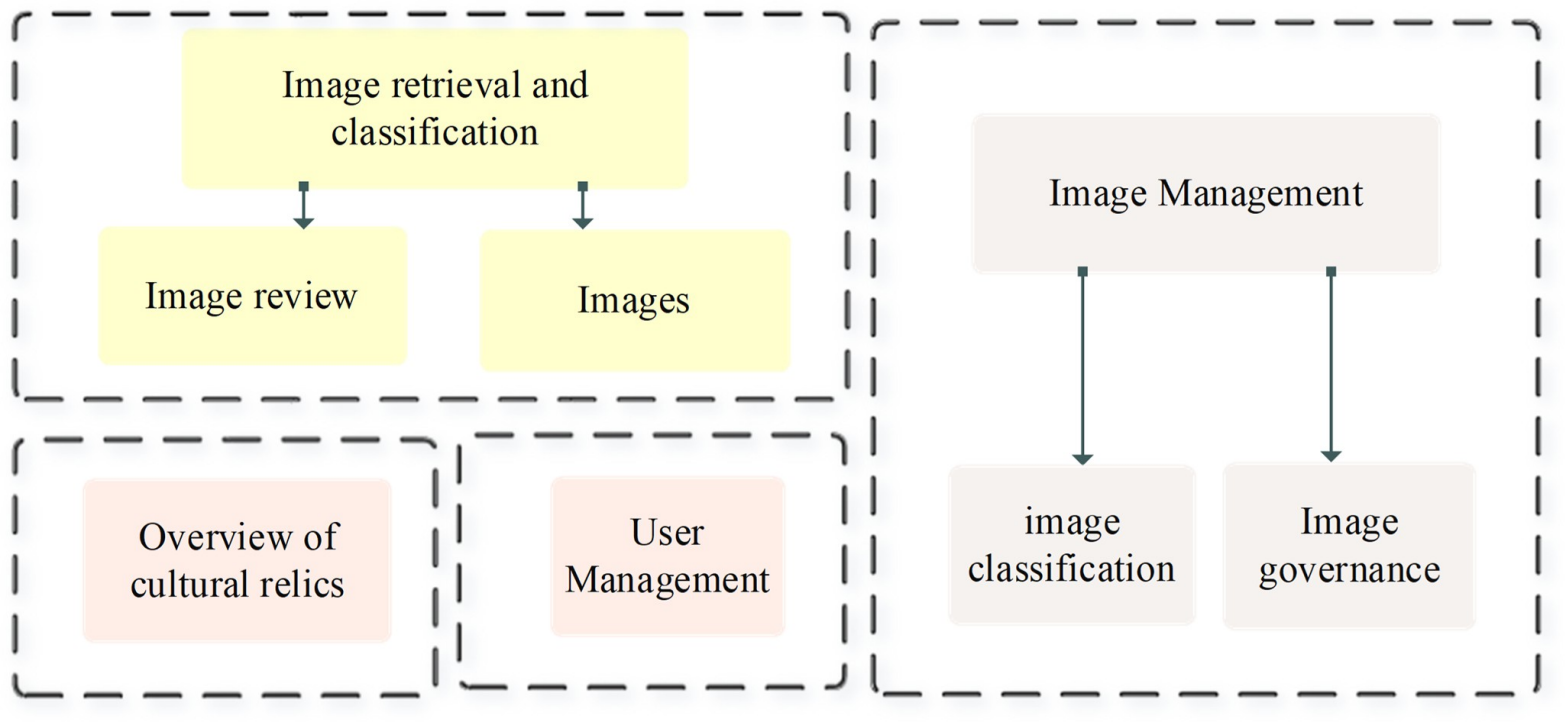
Overall framework of cultural relics image retrieval system.

From the perspective of image data governance, the function of the entire cultural relics image retrieval system is designed [18–20]. The design covers four key modules: image management, overview of cultural relics, user management, and image retrieval and classification. The overall frame diagram of the cultural relics image retrieval system is presented in Fig 3.

In the overview of cultural relics module, the focus is on in-depth statistical analysis of rich image data to offer administrators comprehensive data insights. By employing advanced data analysis technology, key feature information in the cultural relics image database can be effectively extracted, involving artistic style, creation time, author, etc. [21]. The results of these analyses are presented in an intuitive visualization that provides the administrator with a holistic view of the cultural relics collection. By analyzing the correlation between different attributes, administrators can better grasp the characteristics and trends of relics collection and offer strong support for future collection strategies and exhibition planning [22–24].

In the experimental phase, a carefully constructed cultural relics images database is adopted to ensure the system’s accuracy and diversity in data support. Such database support provides a real and representative data scenario for the system prototype in this study, to better verify the system’s performance in image data processing and analysis [25]. In the user management module, flexible management of system user rights is realized to meet different user roles’ requirements [26]. The layered architecture ensures that administrators can effectively add, delete, and modify users, thus realizing the comprehensive management of user information. This layered structure helps maintain the system’s security and brings a high degree of control and flexibility to the user management process, ensuring the system’s stable operation and user experience.

As the core part of the system, image management and image retrieval modules deal with the classification and standardization requirements of cultural relics image data. Image retrieval operations allow users to search based on attributes to quickly and accurately find the desired cultural relics image. Meanwhile, image review ensures the quality and accuracy of these image data and the credibility of the entire database [27]. Through the synergy of these two modules, the proposed system can offer efficient image data management and retrieval functions, providing strong support for the digital management of art museums.

**Fig 4:**
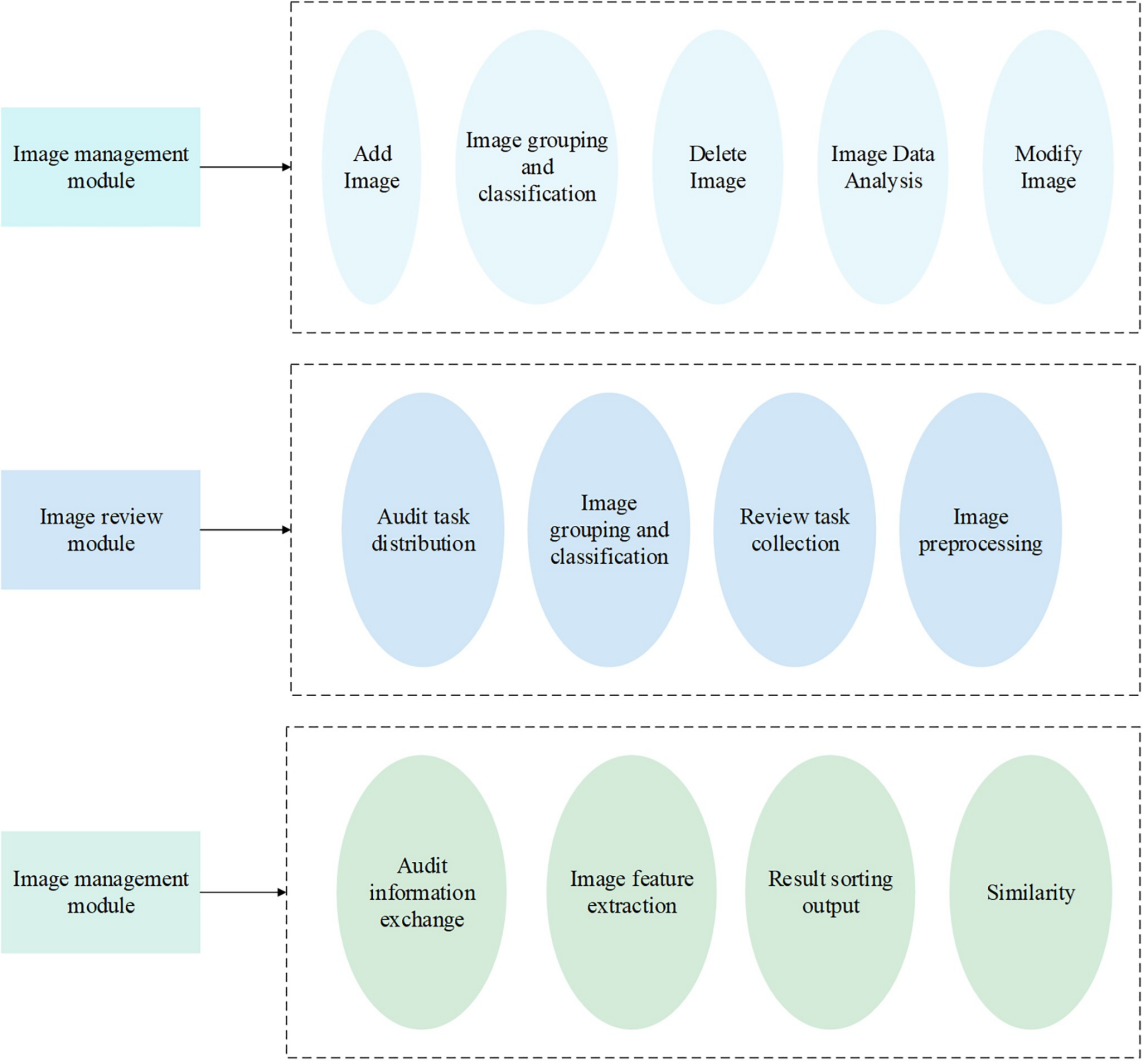
Functional structure of the system.

In addition, according to the specific functional requirements of the system, the functional structure of the cultural relics image retrieval system is constructed, as revealed in Fig 4.

The system’s structure covers three core modules: image management, image retrieval, and image review [28–30]. Among them, the image management module focuses on the grouping and classification of existing image data, and performs data analysis and visual display simultaneously. The image retrieval module is the practical part of the algorithm proposed in this study, including several image preprocessing processes, such as image background segmentation, size standardization, data enhancement, etc. Then, the constructed deep residual network model is used to extract the image features and measure the similarity between the images to be retrieved and the candidate images. Finally, the results are output from high to low in terms of similar scores [31]. The image review module is based on the task mechanism, encompassing task issuance, receiving, and query, to conduct the image matching review. Besides, the system message mechanism strengthens task supervision and progress inquiry among administrators [32].

**Fig 5:**
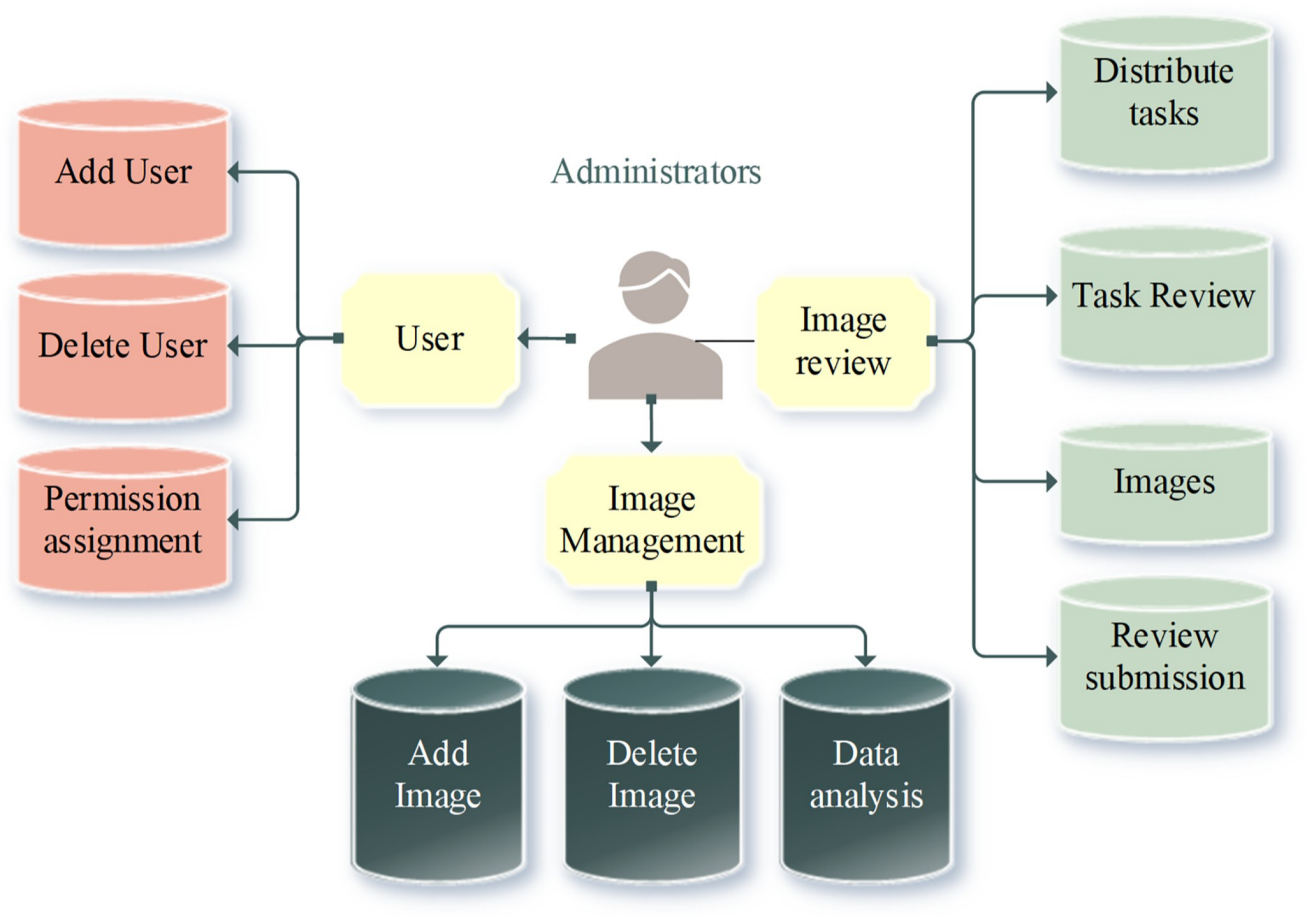
The administrator use case diagram.

This system aims to adapt to the construction of the smart museum and solve the work problems faced by the museum’s internal managers. Therefore, the system’s main role is as an administrator within the museum. The administrator use case diagram is suggested in Fig 5.

To adapt to the construction of smart museums, the system is designed and developed to solve the work problems of internal management personnel of museums. Hence, the system divides multiple administrator roles for different museum managers and specifies various operation authority scopes for each role according to their diverse permissions [33–35]. This differentiated permission setting is designed to guarantee the security and effectiveness of the system. The main operation areas covered by the system include but are not limited to basic management and permission allocation of users, image data management, and data analysis.

These operational areas are a vital part of the system’s functionality, affording administrators the tools and means to effectively manage and operate the museum [36, 37]. In the system, each administrator role assigns tasks to sub-administrators according to the nature and requirements of the task. Sub-administrators can perform image matching and classification operations through the system’s image retrieval function according to the assigned tasks. Specifically, sub-administrators can receive tasks to quickly find matching images and perform reasonable classification by using image retrieval functions, thus completing image review work. The combination of task allocation and image retrieval function can improve the efficiency of image review and ensure the accuracy and reliability of the image review process.

### Composition of the dataset

This paper utilizes image data from the national digital museum cluster "Museum China" website as the data source. Through means such as web crawlers, a self-built cultural relic dataset is created, comprising 3 major categories and 11 subcategories, including bronzeware, pottery, and porcelain, with 400 artifacts in each major category. There are approximately four images per artifact, resulting in a total of 4,780 original images. The composition of the dataset is shown in Table 1.

**Table 1:** The composition of the dataset.

Category	Name	Number of Images
bronze ware	Bronze Tableware	365
	Bronze Wine Vessels	435
	Bronze Water Vessels	380
	Bronze Musical Instruments	410
Chinese Porcelain	Hard Porcelain	545
	Soft Porcelain	490
	Bone China	550
Chinese Pottery	Earthenware Jar	390
	Earthenware Pot	400
	Earthenware Cup	420
	Tang Tri-colored Glazed Pottery	395

Special Note: The author does not own the copyright of the content. According to the copyright statement of “Museum China”, the website strictly adheres to the *Copyright Law of the People’s Republic of China*, the *Regulation on the Protection of the Right to Information Network Dissemination*, and related laws, regulations, and policies. In terms of intellectual property rights, all content published on this website, including but not limited to text, images, audio, and video, belongs to the China Cultural Relics Information Center and the respective museums unless otherwise specified. Individuals and research institutions may freely use the cultural and museum resources on the website for purposes such as education, research, and cultural promotion, provided proper attribution is given. Throughout the course of this study, the research strictly adheres to the terms and conditions of the cited data sources, conducting the collection and analysis of data in a lawful and compliant manner. Before initiating any data retrieval steps, careful consideration is given to and full understanding is reached regarding the usage license agreements, privacy policies, and all applicable legal provisions of the relevant datasets.

### Experimental data design

The experimental platform processor is Intel Core (TM) i7-8700 CPU @ 3.20GHz, and the operating system is 64-bit Windows 10. The client environment is Java Software Engineering (SE) development kit 7, NET framework 4.5.2, C++ 2010, Python3.6. Among them, the GA module of the experimental platform is rewritten based on the open-source package Jpag. The mutation algorithm, fitness calculation, and cross algorithm call the Mutation, Sim, and Cross functions; The CNN module is called the Trainlm function of the Levenberg-Marquardt (L-M) optimization algorithm. The sample data is first normalized during the experiment, and the data results are denormalized. The main parameters of GA are configured as 0.1; The cross-mutation probabilities are 0.3 and 0.1, respectively; The evolutionary algebra is 80. The learning efficiency value of the CNN is 0.9, the dynamic parameter is 0.7, the allowable error condition is Dt 0.00001, and the number of iterations is 100. This study uses the image data of the national digital museum cluster "Museum of China" site as the data source. Moreover, it builds its cultural relics dataset through a web crawler and other means, comprising 3 categories: bronzes, pottery, and porcelain and 11 subcategories. There are 400 kinds of cultural relics in each category, each with about 4 images, for a total of 4780 original images. Although more hidden layer increases the processing power of the neural network, it also brings the adverse result of complicated calculation and long time. Thus, this study adopts the three-layer network structure of the input, hidden, and output layers for simulation. In terms of determining the number of nodes in each layer, 26 evaluation indicators are used as the number of nodes in the input layer, and one node in the aggregate quality evaluation result is selected as the element of the output layer, corresponding to 26 input layer units. The hidden layer has more nodes, and the number of hidden layer nodes is finally determined to be 17.

## Optimal simulation study of digital retrieval system of the art museum

### Analysis of training results of the GA2CNN model

**Fig 6:**
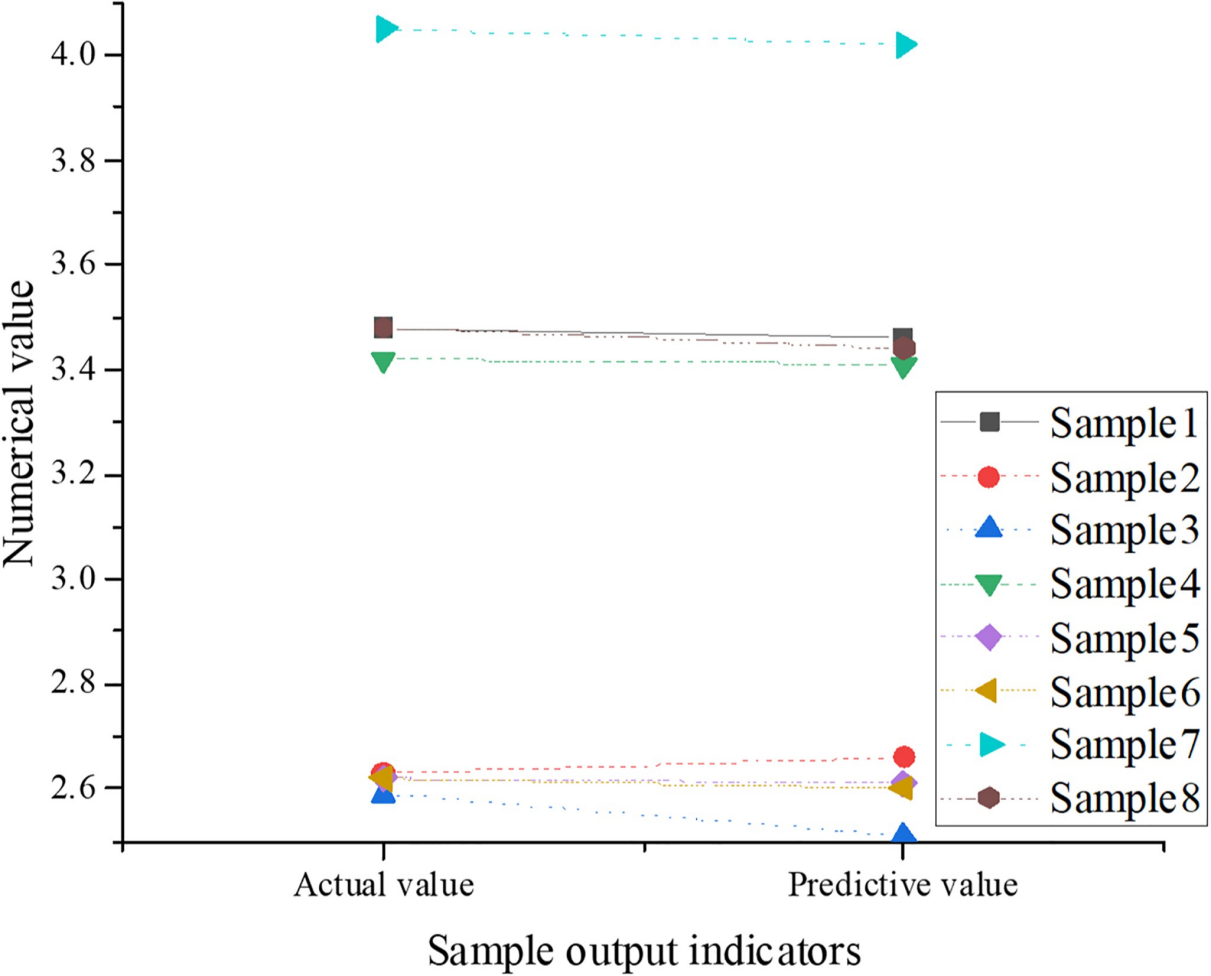
The output of the training sample of the GA2CNN model.

In the process of optimizing the digital art museum retrieval system, the GA2CNN model is trained and its results are analyzed. In the GA2CNN model, samples numbered 1 to 8 are selected as inputs for training the network. Here, the actual values refer to the real numerical values or labels of specific samples in this system, used to train and evaluate the model’s accuracy. In the given dataset, each sample has a corresponding actual value, representing the true situation or attributes of that sample. Data numbered 1 to 8 are chosen as inputs for training the network. Each sample typically contains one or more features describing the attributes or characteristics of that sample. In the research of the digital art museum retrieval system, samples are feature vectors or images of digitized artworks. The output of the training sample of the GA2CNN model is plotted in Fig 6.

The actual values refer to the numerical values corresponding to samples numbered 1 to 8 under actual conditions. The corresponding actual values are 3.48, 2.63, 2.59, 3.42, 2.62, 2.62, 4.05, and 3.48. The predicted values are the values predicted by the GA2CNN model based on the input sample data after training. The corresponding predicted values are 3.46, 2.66, 2.51, 3.41, 2.61, 2.6, 4.02, and 3.44. The error values represent the differences between actual and predicted values. The calculation method involves subtracting the predicted value of each sample from its corresponding actual value to obtain the error value. The corresponding error values are 0.02, 0.03, 0.08, 0.01, 0.01, 0.02, 0.03, and 0.04. These data represent the GA2CNN model’s performance and accuracy. By comparing actual values with predicted values, the model’s predictive ability can be observed. The model’s actual values and predicted values are very close, with absolute errors ranging from 0.01 to 0.1, indicating a relatively small average error and high model prediction accuracy.

**Fig 7:**
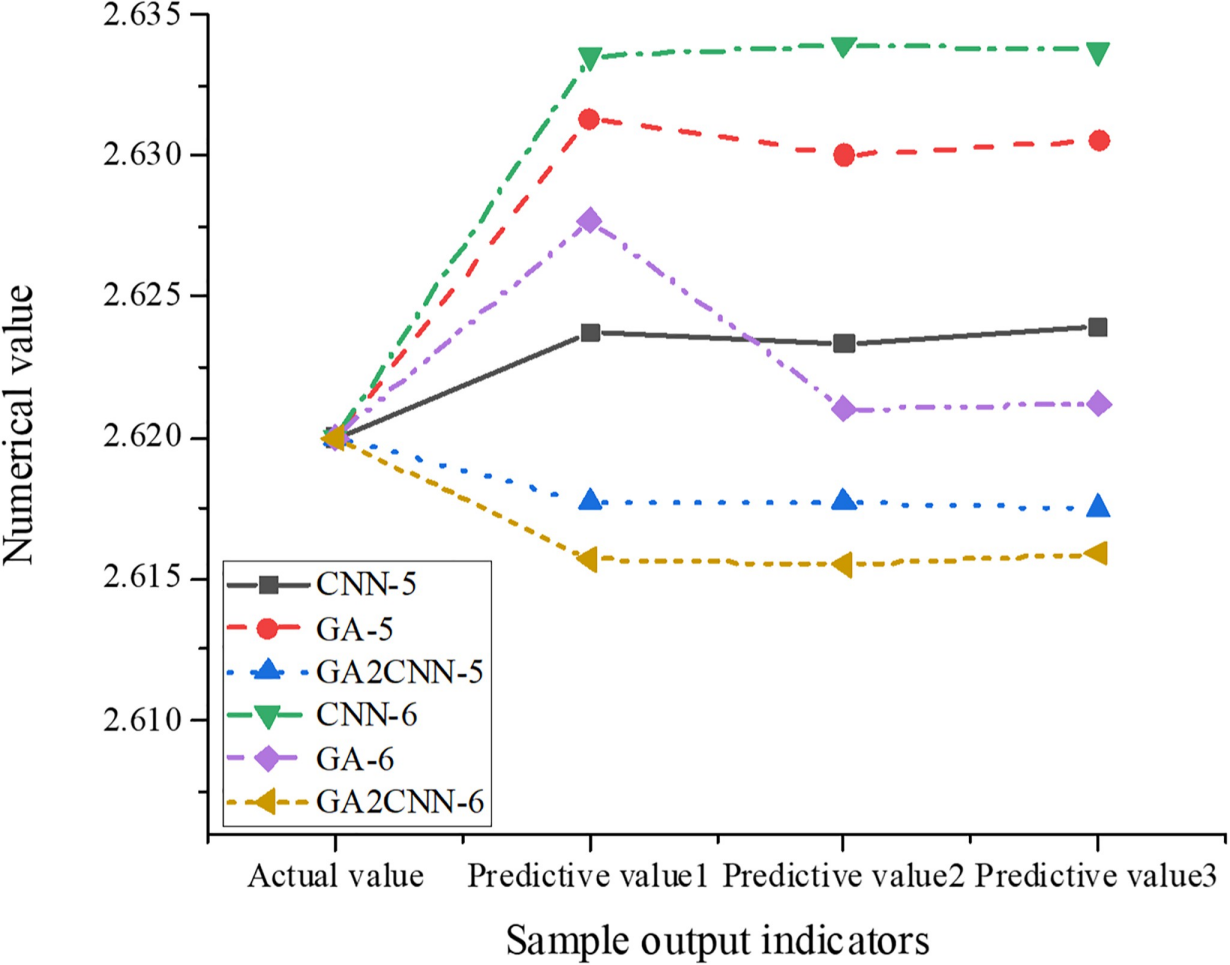
Comparison of the output results of three models.

To test the GA2CNN model’s effectiveness and better reflect its superiority, the data with samples 5 and 6 are input into the model, and the calculation results are output. At the same time, the predicted values of the detected data are compared by GA and CNN. The comparison of the output results of the three model detection samples is illustrated in Fig 7.

Fig 7 signifies the calculation results of the data with samples 5 and 6 under various prediction models, including GA, CNN, and GA2CNN models. In all models, the actual value of the sample output is 2.62, demonstrating the real data observation. For the results of the first prediction of sample 5, compared with the actual value of 2.62, the GA2CNN model’s predicted value is 2.6177, with an error of 0.0023; The CNN and GA models’ predicted values are 2.6237 and 2.6313, with an error of 0.0037 and 0.0113. For the first prediction result of sample 6, compared with the actual value of 2.62, the GA2CNN, GA, and CNN models’ predicted values are 2.6157, 2.6277, and 2.6335, and their error values are 0.0043, 0.0077, and 0.0135. It can be found that the optimized GA2CNN model’s network fitting accuracy is high, the predicted value is very close to the actual value, and the digital retrieval system integrated into the GA2CNN model performs well in improving retrieval efficiency and accuracy.

Then, all samples are input to obtain the output results, as exhibited in Table 2.

**Table 2:** The output of the three models on all samples.

Sample number	Actual value	Predictive value	Error
1	2.62	2.6237	0.0037
2	2.62	2.6313	0.0113
3	2.62	2.6177	0.0023
4	2.62	2.6241	0.0041
5	2.62	2.6287	0.0087
6	2.62	2.6265	0.0065
7	2.62	2.6273	0.0073
8	2.62	2.6290	0.009

According to the data shown in Table 1, the output results of the three models on all samples have been listed. The mean is approximately 2.6264, and the standard deviation is about 0.0028, indicating that the model’s predictions are generally accurate and have some stability. From the error column, it can be seen that the average error between predicted and actual values is relatively small, with errors mostly below 0.01 for most samples. This shows that the model’s predictive ability is fairly reliable, although there are a few samples with larger errors, suggesting that further optimization of the model may be needed to improve accuracy. Overall, these results suggest that the established model has a certain feasibility and accuracy in predicting actual values.

### Retrieval effect analysis of digital retrieval system by integrating intelligent information and improved GA

**Fig 8:**
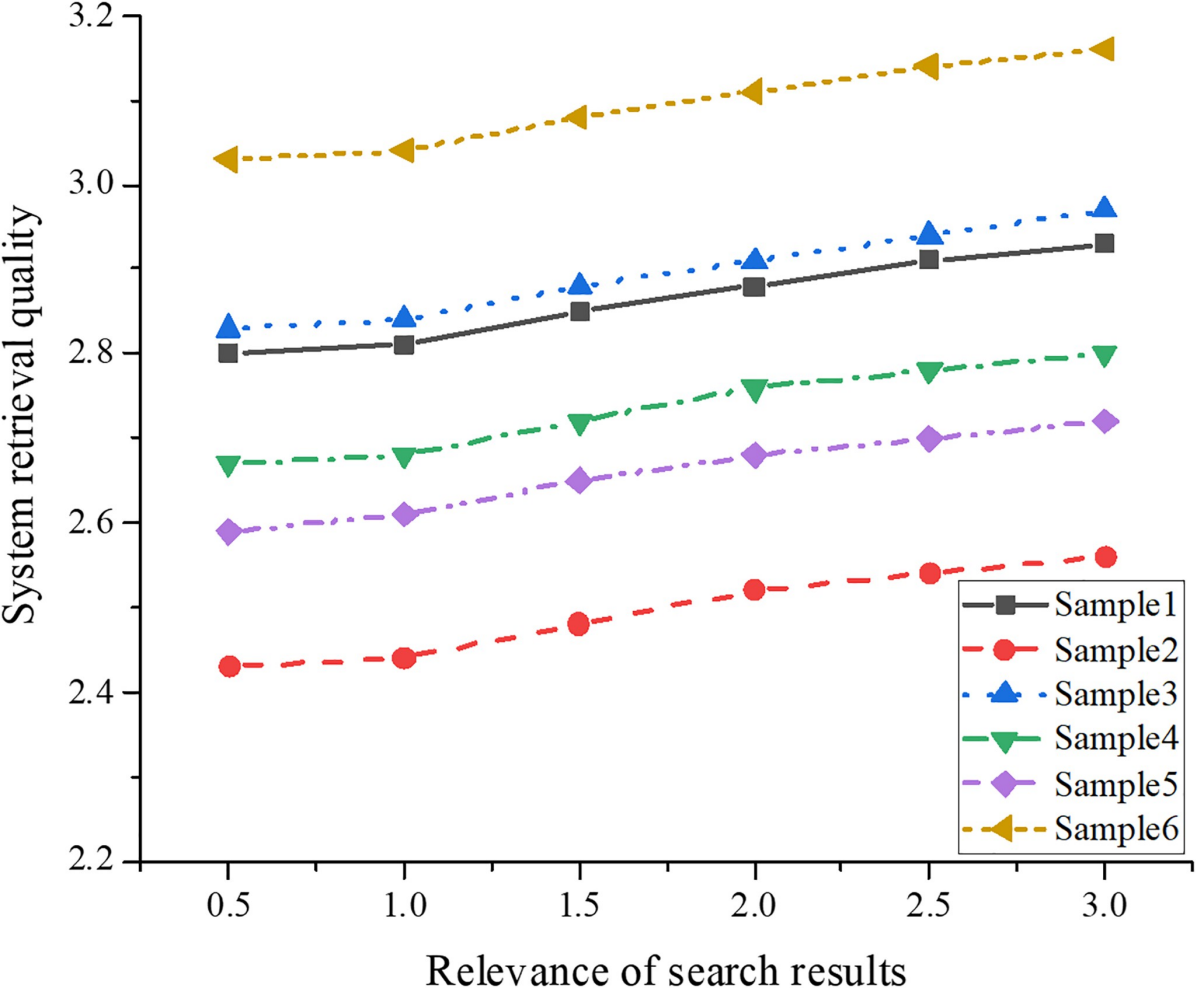
Retrieval effect of digital retrieval system on different samples.

To investigate the influence of changes in various samples and relevance index values on the digital retrieval system, this study inputs the predicted data into the computer. Moreover, this study also obtains the retrieval effect of the digital retrieval system on different samples, as portrayed in Fig 8.

With the gradual increase of the retrieval relevance index value, the retrieval effect value of the digital retrieval system on diverse samples presents the following trend. For sample 1, as the index value gradually adds from 0.5 to 3, its retrieval effect value gradually enhances. When the index value rises from 0.5 to 3, sample 1’s effect value increases from 2.8 to 3.03. Similarly, sample 2’s effect value gradually increases as the retrieval relevance index rises. For instance, when the retrieval relevance index value is 0.5 and 3, the effect value of sample 2 is 2.81 and 3.16. A similar trend can be observed in samples 3 to 6. With the rise of the retrieval relevance index value, these samples’ retrieval effect value is gradually improved. Taking sample 3 as an example, when the index value increases from 0.5 to 3, its retrieval effect rises from 2.43 to 3.08. To sum up, in this dataset, no matter which sample, with the increase of the retrieval relevance index value, its effect value shows a trend of gradual improvement. This means that under the requirement of higher relevance, the proposed system can match and return the results related to the user’s query more accurately, thus promoting the retrieval quality of the system.

The research findings above indicate that, across different samples, as the relevance index values for retrieval gradually rise, the retrieval effectiveness values of the digital retrieval system also show a gradual improvement trend. Specifically, as the relevance index values improve from 0.5 to 3, the retrieval effectiveness values for various samples increase. This phenomenon reflects the proposed system’s performance and retrieval quality under different relevance requirements. Firstly, when the relevance index value is low (such as 0.5), this system may return results with low relevance to the user query keywords, resulting in relatively low retrieval effectiveness values. This suggests that the system may have some errors or inaccuracies in matching under such circumstances, affecting the quality of the retrieval results. As the relevance index values increase, the system can more accurately match user queries, returning results more relevant to the query keywords. Therefore, as the relevance index values rise from 0.5 to 3, the retrieval effectiveness values also gradually increase. It indicates an improvement in system performance, and users can obtain retrieval results more in line with their expectations. This trend has been validated across diverse samples, illustrating that the improvement in the system performance is universal, not limited to a specific dataset or sample. This affords vital reference and guidance for optimizing and improving the digital retrieval system, which can enhance retrieval quality and user experience by increasing relevance index values.

## Conclusion

Against the background of art museum digital management, this study discusses the development of a digital retrieval system that integrates intelligent information processing technology and improved GA. A GA2CNN optimization model combining domain knowledge is proposed to optimize search ranking to better match users’ search intentions. This study constructs the function modules of cultural relics overview, user management, image management, and image retrieval in the system design and implementation stage. Through the realization of image feature extraction, text description generation, and review function, an effective means is provided for the processing and standardizing of cultural relics image data. In addition, applying intelligent information processing technology enables users to express their search needs more accurately and improves the quality of search results. The introduction of improved GA further optimizes the ranking of image retrieval and offers search results that align with users’ interests. Through the analysis of the experimental results, it is found that with the gradual improvement of the system’s retrieval relevance index value, the retrieval effect value of the digital retrieval system on different samples presents the following trend. For sample 1, as the retrieval relevancy index value gradually increases from 0.5 to 3, its retrieval effect values are 2.8 and 3.03, indicating its retrieval effect value gradually improves. In sample 2, similarly, with the rise of the index value, the search effect value exhibits a trend of gradual improvement, thus confirming the application potential of the system in the art museum field.

This study develops a digital retrieval system by integrating intelligent information processing technology and improved GA. In addition, it conducts empirical research in the field of art museums. The main findings are as follows. (1) The digital retrieval system integrating intelligent information processing technology and improved GA can markedly improve retrieval effectiveness and user experience. (2) Experimental results demonstrate that under different relevance requirements, the system’s retrieval effectiveness values gradually increase, proving the superiority of the system in matching and returning results relevant to user queries. (3) This study provides new ideas and methods for the digital retrieval system’s application in the art field, offering beneficial references for research and practice in related fields. Future research directions include: (1) Further optimization of algorithms: Algorithms combining intelligent information processing technology and improved GA can be further improved to enhance system performance and efficiency. (2) Expansion of application areas: The digital retrieval system can be expanded to other fields such as literature and history, exploring its application potential in diverse domains. (3) Optimization of user experience: The system’s user interface and interaction design can be further optimized to enhance user retrieval experience and satisfaction. Through further research and practice, the digital retrieval system can be continuously improved to play a greater role in applications in art museums and other fields, providing users with better services and experiences.

## Supporting information

S1 Data (ZIP)
