# Supplementary material for: Development of the digital retrieval system integrating intelligent information and improved genetic algorithm: A study based on art museums
Source: PLoS One. 2024 Jun 25;19(6):e0305690. doi: 10.1371/journal.pone.0305690 (PMC11198836; doi:10.1371/journal.pone.0305690)
Supplement: S1 Data — (ZIP) [file pone.0305690.s001.zip › data packet/Code Description.docx]

The code imports three modules (`image_processing`, `natural_language_processing`, and `genetic_algorithm`) and establishes a database connection. It defines classes and functions to build the fundamental framework of a digital retrieval system. Here's a description of the code:

1. At the beginning of the code, three modules are imported: `image_processing`, `natural_language_processing`, and `genetic_algorithm`. These modules likely contain functions and methods related to image processing, natural language processing, and genetic algorithms, respectively, which will be utilized for subsequent system functionalities.

2. The code establishes a database connection through the line `database = connect_to_database()`. The database is expected to serve as a storage place for various system data such as image data and user information.

3. The function `extract_features(image)` is designed to extract features from an image. It starts by calling `image_processing.extract_features(image)` to retrieve the image's feature information. Then, it employs `natural_language_processing.generate_description(features)` to create a text description of the image based on the extracted features. Eventually, it returns both the extracted features and the generated text description.

4. Following this, three classes are defined: `UserManager`, `ImageManager`, and `ImageRetrieval`.

- The `UserManager` class comprises three methods: `add_user`, `delete_user`, and `modify_user`. Although these methods are currently not implemented, they are anticipated to manage user operations such as adding, deleting, and modifying users.

- The `ImageManager` class encompasses two methods: `upload_image` and `audit_image`. The `upload_image` method takes an image as input, extracts image features and descriptions using the `extract_features` function, and then inserts this information into the database. The `audit_image` method updates the image's status in the database based on the outcome of the image audit.

- The `ImageRetrieval` class defines a method `search_images` that accepts query parameters. It uses the `natural_language_processing` module to parse the query and then employs the `genetic_algorithm` module to execute optimized image retrieval ranking. Finally, it returns the ranking results.

5. Under the `if __name__ == "__main__":` statement, three instances are created: `user_manager`, `image_manager`, and `image_retrieval`. These instances can be used to call the methods defined in their respective classes, thereby implementing the functionalities of the digital retrieval system.

6. Demonstrations are provided using the lines `user_manager.add_user("Alice")`, `user_manager.add_user("Bob")`, and `user_manager.add_user("Carol")`. These lines illustrate how the `add_user` method of the `UserManager` class is utilized to add different users.

7. The line `user_manager.delete_user("Bob")` showcases how the `delete_user` method of the `UserManager` class is used to delete the user "Bob".

8. The line `user_manager.modify_user("Alice")` demonstrates how the `modify_user` method of the `UserManager` class is employed to modify the information of the user "Alice".

9. The line `image_manager.upload_image(image)` illustrates how the `upload_image` method of the `ImageManager` class is used to upload an image. This method automatically extracts features and descriptions from the image, and then stores the image along with the information in the database.

10. Using the line `image_manager.audit_image(1)`, the code showcases how the `audit_image` method of the `ImageManager` class is used to audit an image. The status of the image in the database is updated based on whether the image passes the audit.

11. Lastly, the line `image_retrieval.search_images(query)` demonstrates how the `search_images` method of the `ImageRetrieval` class is employed for image retrieval. The query is parsed using natural language processing, and the image ranking is optimized using genetic algorithms.
