# Supplementary figures and images for: Development of the digital retrieval system integrating intelligent information and improved genetic algorithm: A study based on art museums
Source: PLoS One. 2024 Jun 25;19(6):e0305690. doi: 10.1371/journal.pone.0305690 (PMC11198836; doi:10.1371/journal.pone.0305690)

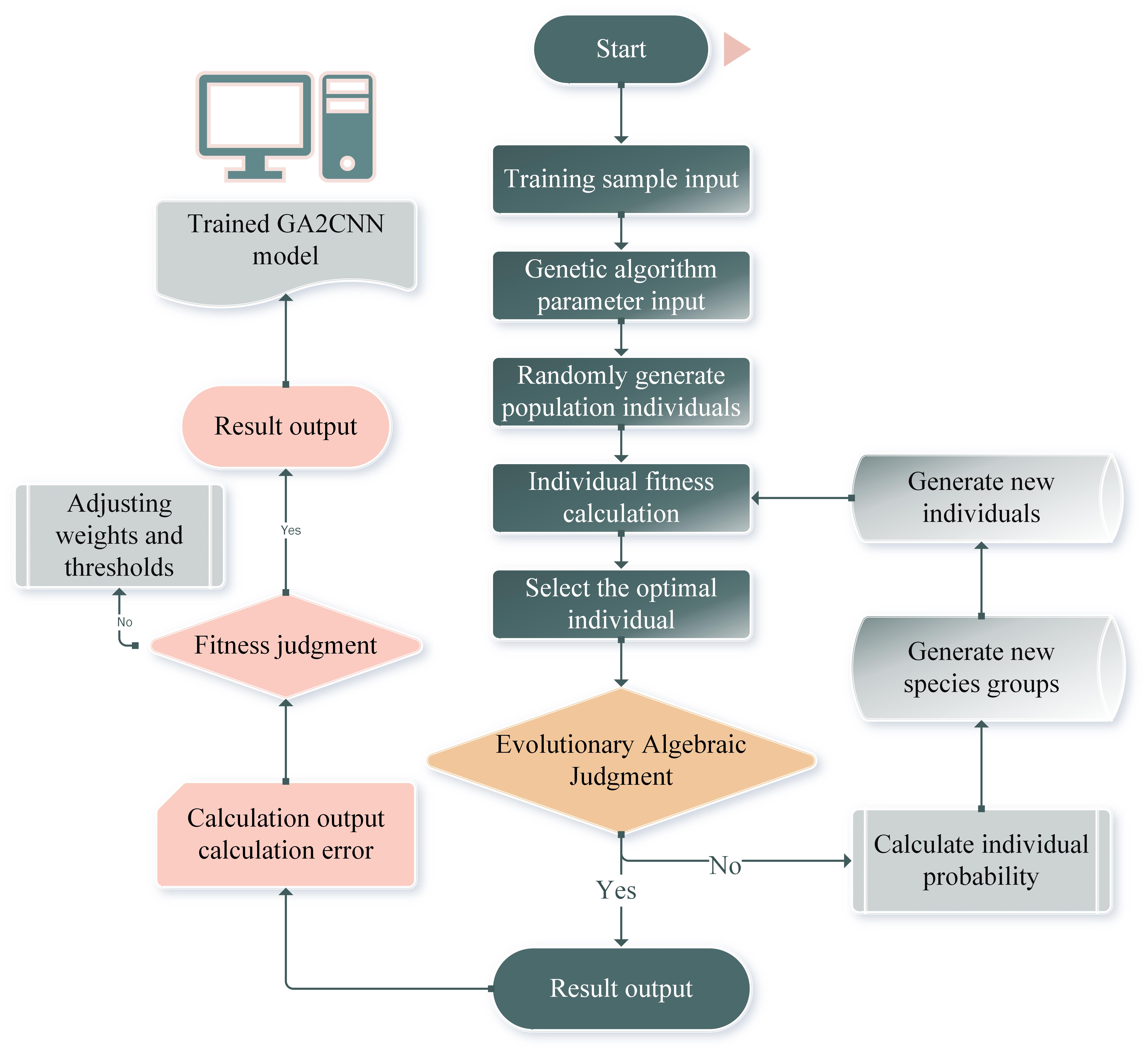

Supplement: S1 Data — (ZIP) [file pone.0305690.s001.zip › data packet/Figure1.jpg]

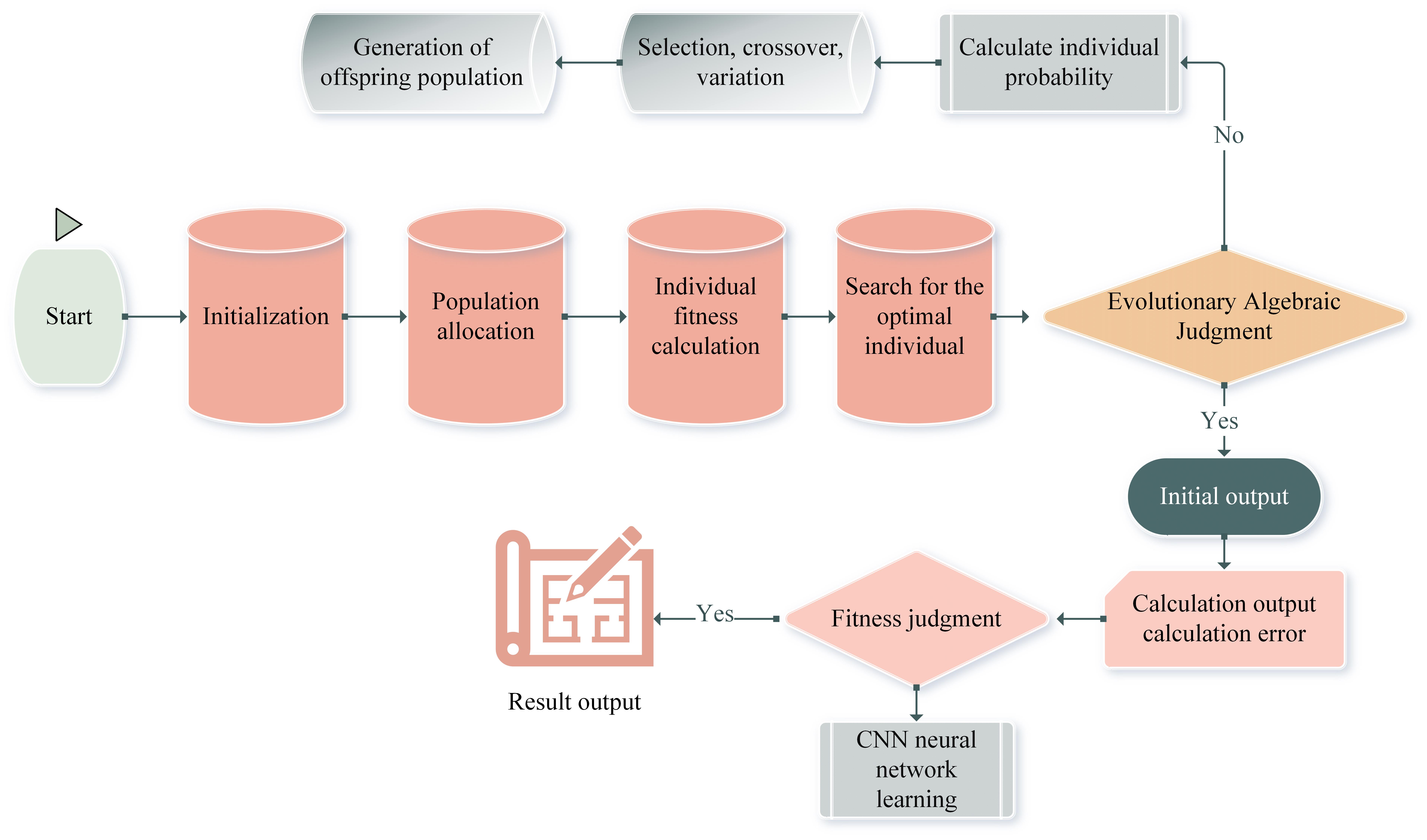

Supplement: S1 Data — (ZIP) [file pone.0305690.s001.zip › data packet/Figure2.jpg]

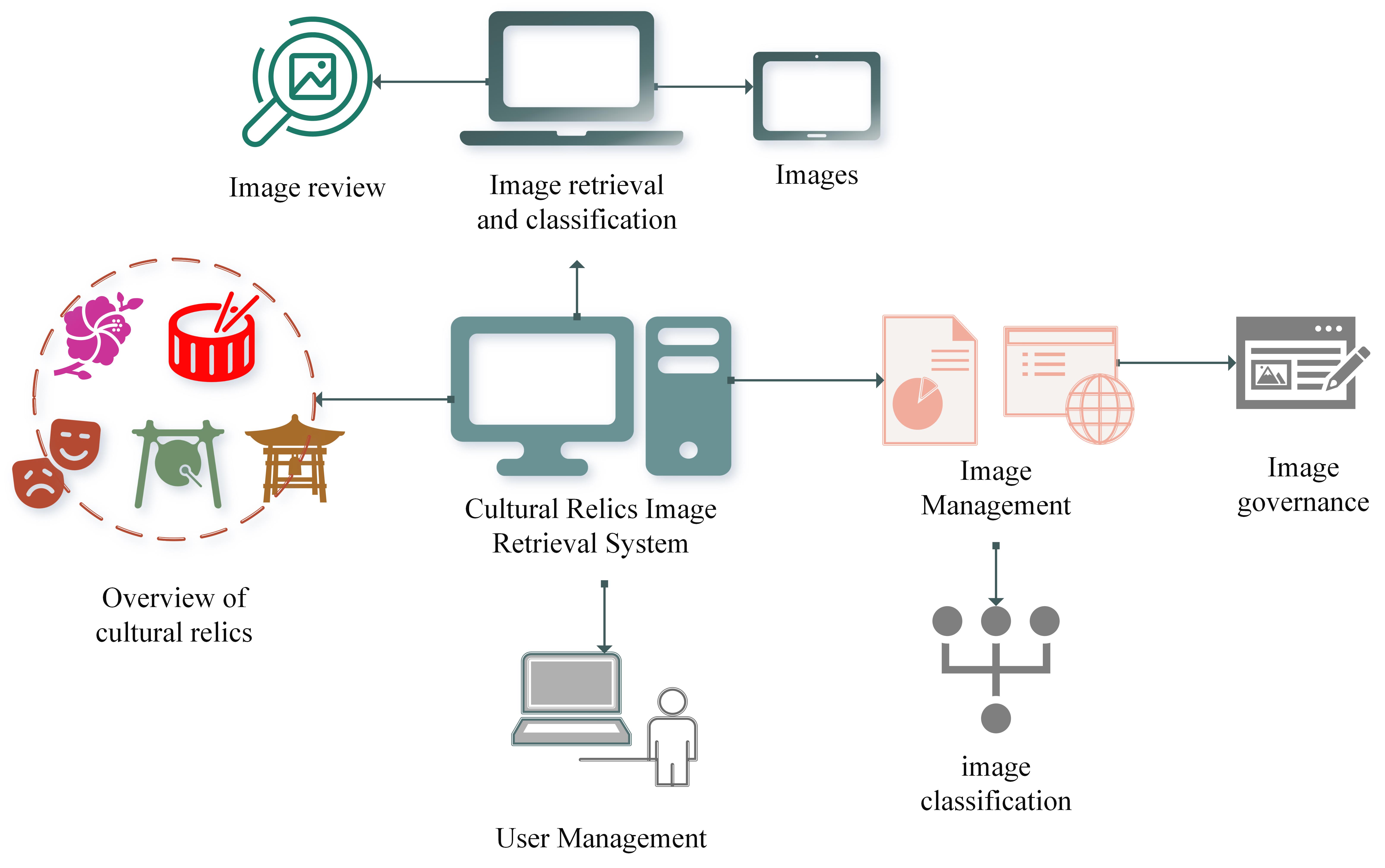

Supplement: S1 Data — (ZIP) [file pone.0305690.s001.zip › data packet/Figure3.jpg]

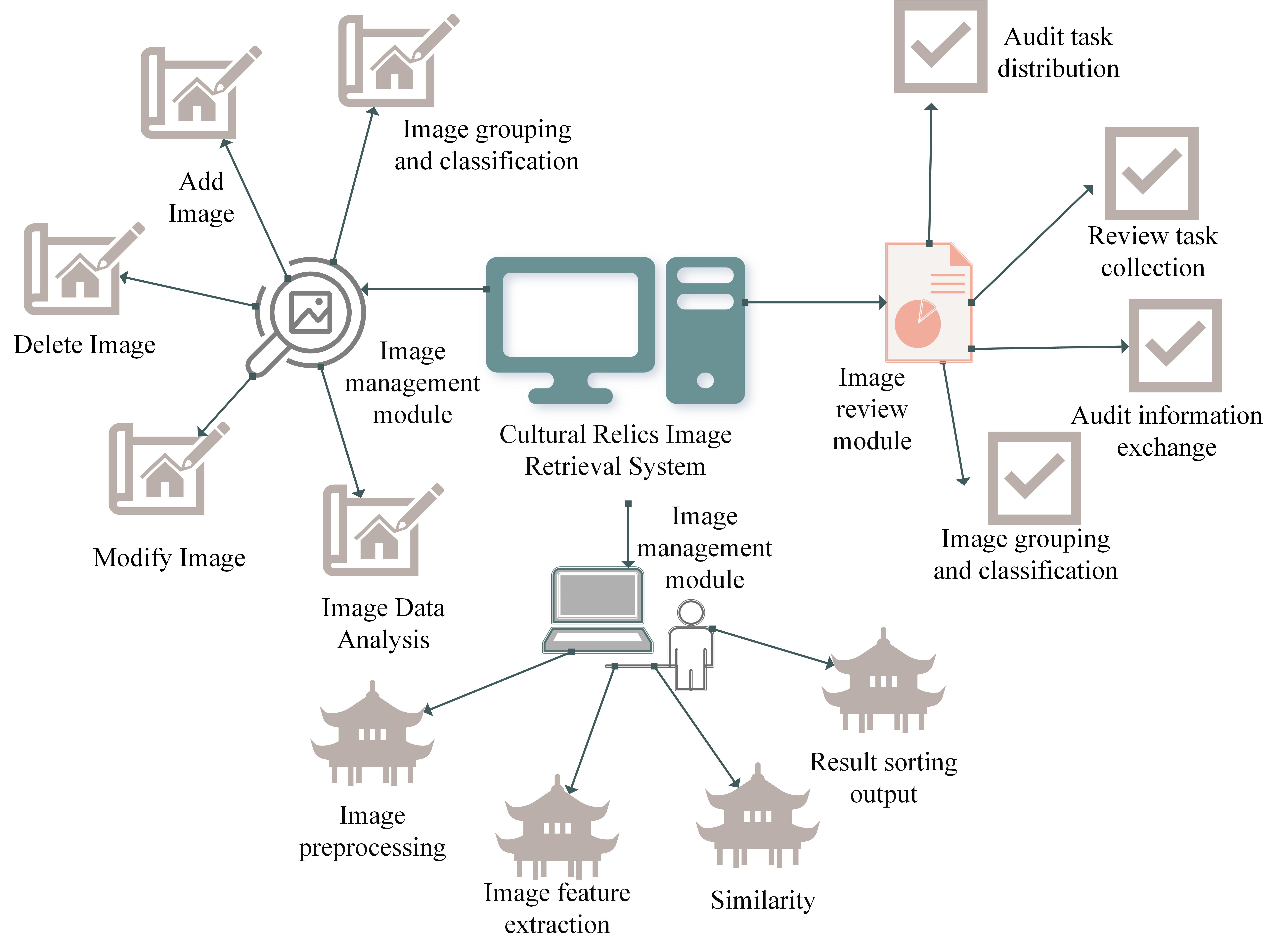

Supplement: S1 Data — (ZIP) [file pone.0305690.s001.zip › data packet/Figure4.jpg]

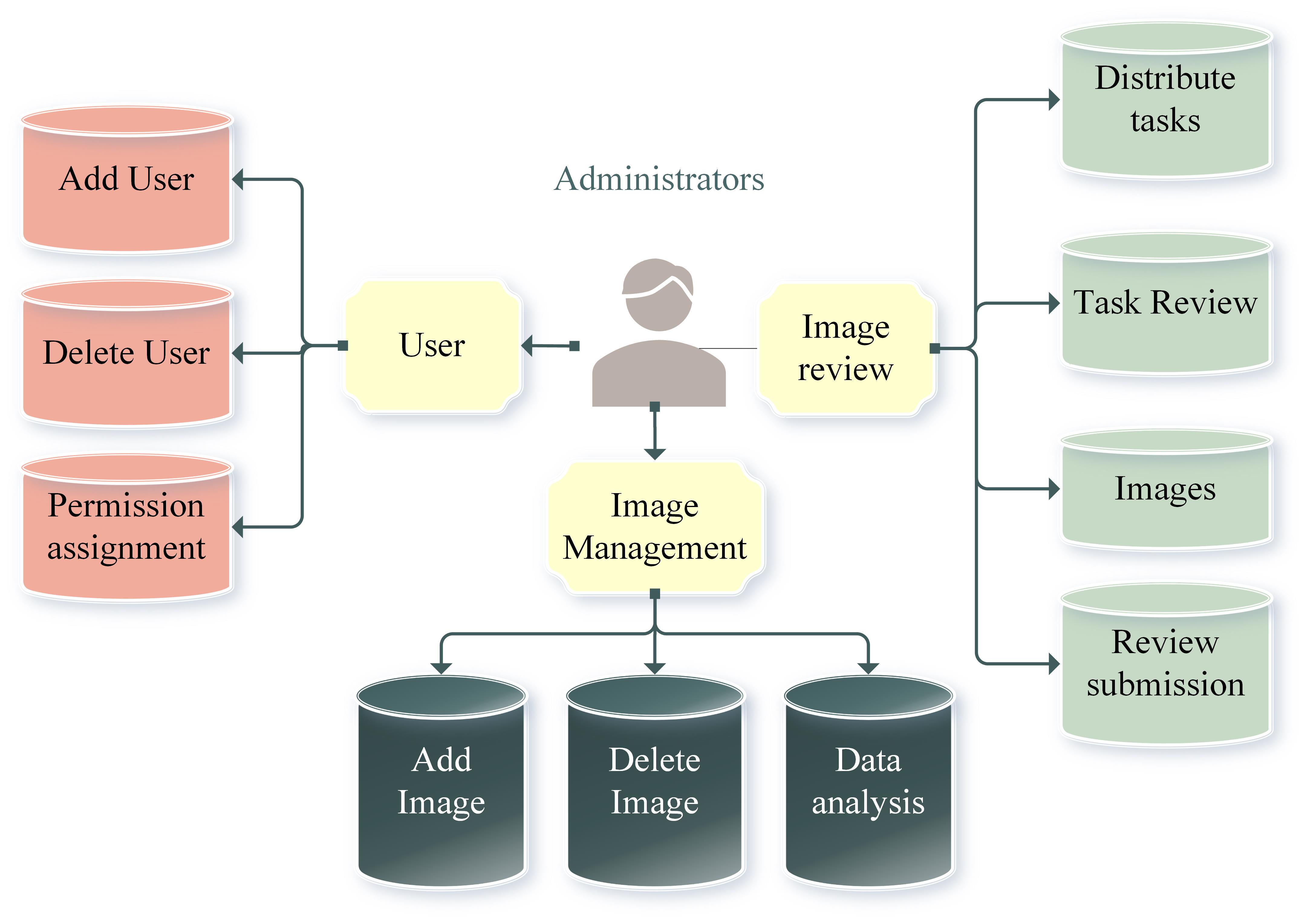

Supplement: S1 Data — (ZIP) [file pone.0305690.s001.zip › data packet/Figure5.jpg]

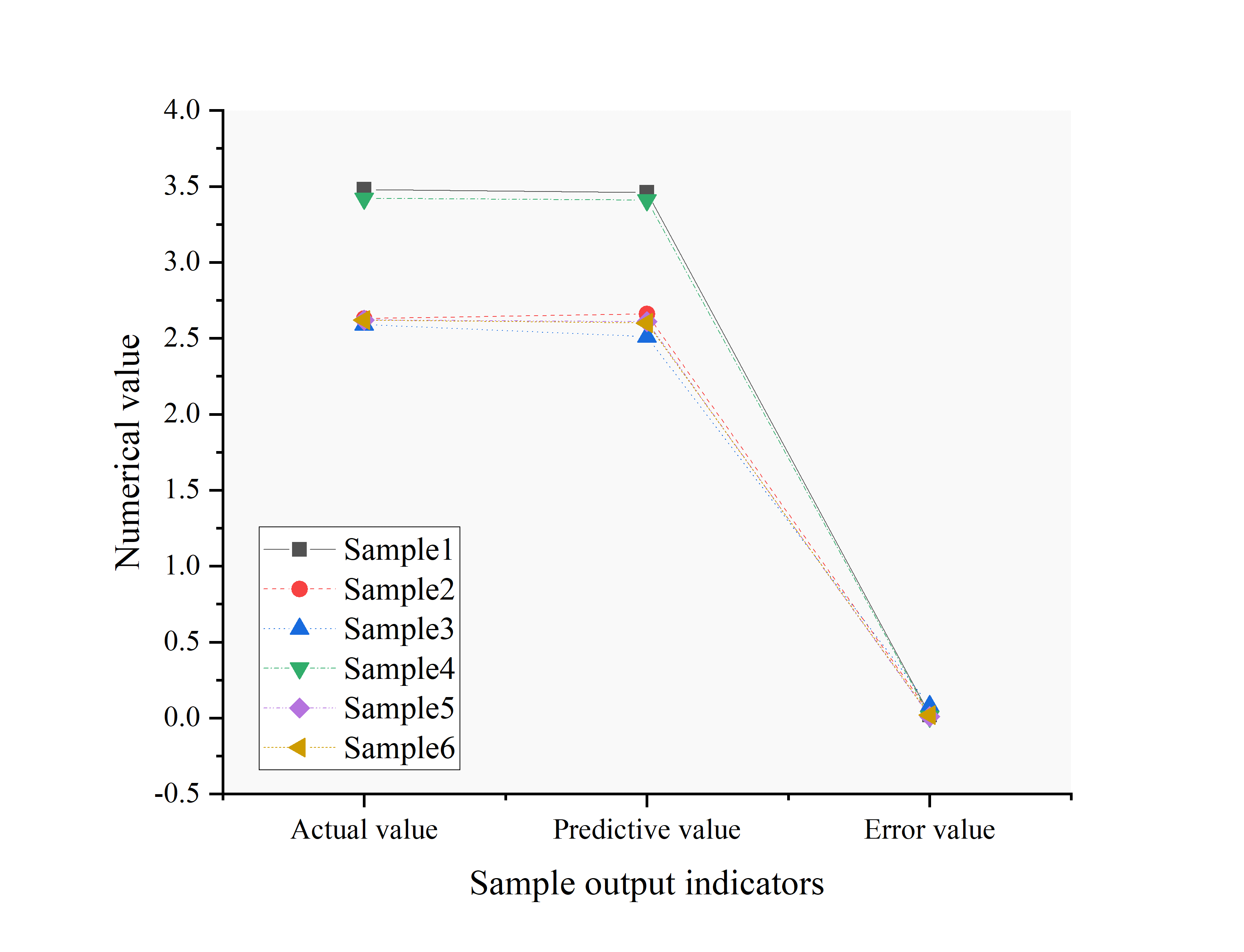

Supplement: S1 Data — (ZIP) [file pone.0305690.s001.zip › data packet/Figure6.png]

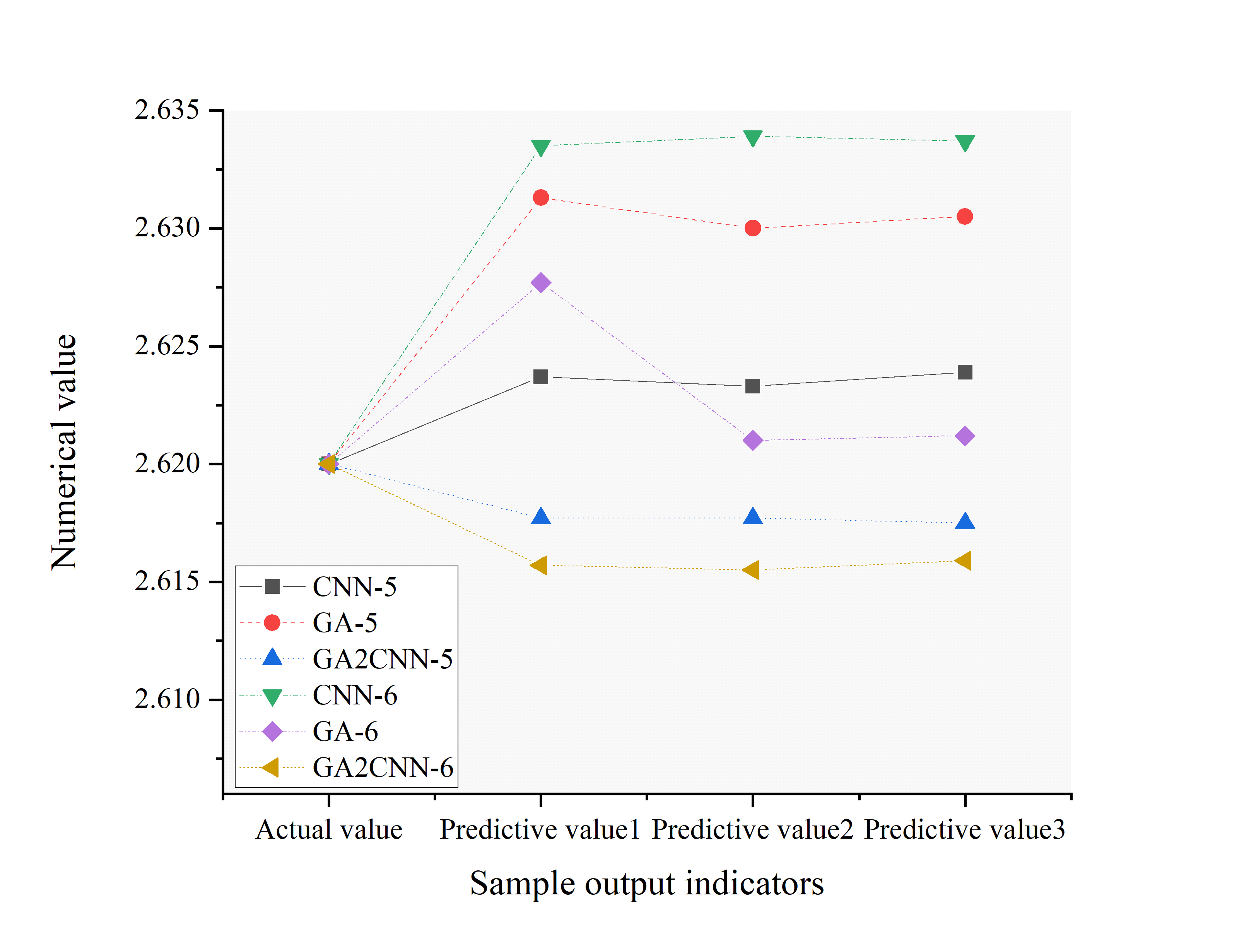

Supplement: S1 Data — (ZIP) [file pone.0305690.s001.zip › data packet/Figure7.png]

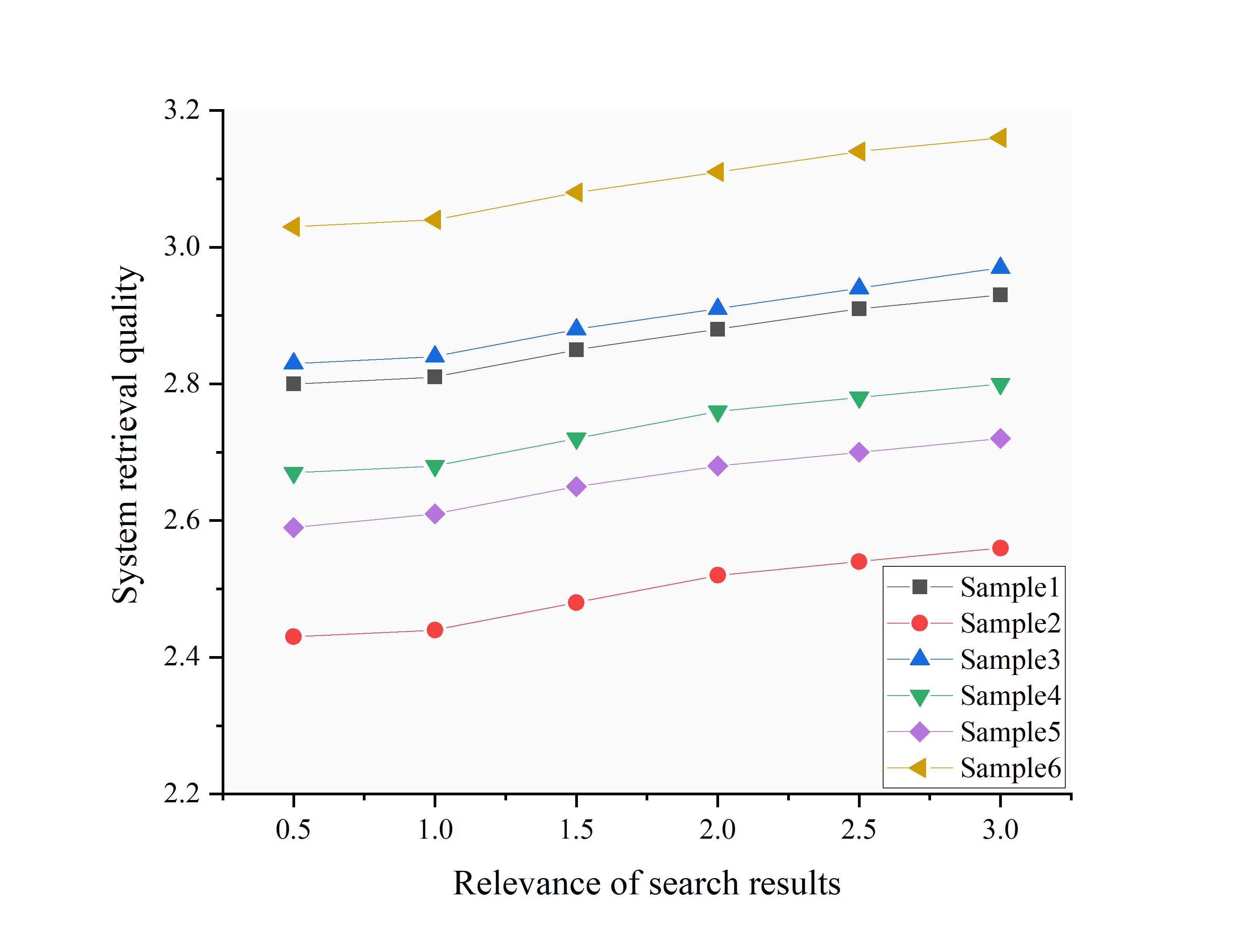

Supplement: S1 Data — (ZIP) [file pone.0305690.s001.zip › data packet/Figure8.png]
